# Markers of Intestinal Permeability Are Rapidly Improved by Alcohol Withdrawal in Patients with Alcohol-Related Liver Disease

**DOI:** 10.3390/nu13051659

**Published:** 2021-05-14

**Authors:** Finn Jung, Katharina Burger, Raphaela Staltner, Annette Brandt, Sebastian Mueller, Ina Bergheim

**Affiliations:** 1 Department of Nutritional Sciences, Molecular Nutritional Science, University of Vienna, 1090 Vienna, Austria; finn.jung@univie.ac.at (F.J.); katharina.burger@univie.ac.at (K.B.); raphaela.staltner@univie.ac.at (R.S.); annette.brandt@univie.ac.at (A.B.); 2 Department of Medicine, Salem Medical Center and Center for Alcohol Research, University of Heidelberg, 69121 Heidelberg, Germany; sebastian.mueller@urz.uni-heidelberg.de

**Keywords:** endotoxin, I-FABP, intestinal barrier, alcohol, liver

## Abstract

Changes in intestinal microbiome and barrier function are critical in the development of alcohol-related liver disease (ALD). Here, we determined the effects of a one-week alcohol withdrawal on parameters of intestinal barrier function in heavy drinkers with ALD in comparison to healthy non-drinkers (controls). In serum samples of 17 controls (m = 10/f = 7) and 37 age-matched ALD patients (m = 26/f = 11) undergoing a one-week alcohol withdrawal, markers of liver health and intestinal barrier function were assessed. Liver damage, e.g., fibrosis and hepatic steatosis, were assessed using FibroScan. Before alcohol withdrawal, markers of liver damage, lipopolysaccharide binding protein (LBP) and overall TLR4/TLR2 ligands in serum were significantly higher in ALD patients than in controls, whereas intestinal fatty acid binding protein (I-FABP) and zonulin protein concentrations in serum were lower. All parameters, with the exception of LBP, were significantly improved after alcohol withdrawal; however, not to the level of controls. Our data suggest that one-week of abstinence improves markers of intestinal barrier function and liver health in ALD patients.

## 1. Introduction

Despite intense public campaigns informing of the risks of high and especially chronic alcohol ingestion, alcohol consumption is still among the leading causes of liver damage in many countries world-wide [1]. Alcohol-related liver disease (ALD) comprises a spectrum of conditions ranging from simple steatosis to alcoholic hepatitis and even advanced fibrosis, as well as cirrhosis [2]. Indeed, it is estimated that ~10–15% of alcoholics will progress to liver cirrhosis [3]. Intense research efforts have been undertaken to delineate molecular mechanisms underlying the development of ALD; however, mechanisms are still not fully understood and therapies primarily focus on abstinence being afflicted with high relapse rate. It has been shown that the development of ALD is associated with changes of intestinal microbiota composition in the upper parts of the small intestine and elevated bacterial endotoxin levels (for overview see [4]), further suggesting that ALD may not only be a disease evolving from the direct effects of ethanol metabolism in the liver, e.g., the increase in the ratio of NADH + H^+^ to NAD^+^ and induction of CYP2E1. Indeed, it has been shown that chronic alcohol intake in patients with liver cirrhosis is associated with a higher prevalence of *Proteobacteria* and lower abundance of *Bacteroidetes* in feces [5]. In the liver, gut-derived bacterial endotoxin has been shown to play a pivotal role in the hypermetabolic state, e.g., increased hepatic oxygen uptake and accumulation of lipids after acute alcohol exposure [6,7]. Interventions, be it genetic or pharmacological, aiming to disrupt the loss of intestinal barrier function or recognition of endotoxin by liver cells in settings of chronic alcohol ingestion have been shown to be associated with a marked protection from the development of ALD in rodents (for overview also see [8]). Furthermore, in settings of alcohol-dependence, it has been shown that abstinence for several weeks is associated with a marked decrease of bacterial endotoxin serum levels [9].

Starting from this background, the present study aimed to determine the effect of a one-week alcohol withdrawal of ALD patients on markers of intestinal barrier function and if this is related to changes of markers of liver-related health.

## 2. Materials and Methods

### 2.1. Subjects

A total of 37 patients with ALD, as diagnosed by ultrasound and Fibroscan [10] and 17 age-matched normal weight healthy non-drinkers (controls) were recruited at the Department of Nutritional Sciences, Vienna, Austria and the Department of Medicine, Salem Medical Center, Heidelberg, Germany. For details of inclusion and exclusion as well as diagnosis of ALD, see Mueller et al. [11]. Only ALD patients aged >18 years with negative serological screening for antimitochondrial and antinuclear antibodies as well as HBV and HCV were included in the study. The ethics committee of the medical faculty of Heidelberg (Germany, reference: S150/2015) and of the Medical University Vienna (Austria; reference: 1366/2017) approved the studies which were carried out in accordance with the Helsinki II Declaration. All patients and controls enrolled gave their written informed consent to participate in the study. Data and fasting blood samples of ALD patients were obtained at the admittance to the alcohol withdrawal unit of Salem Hospital, Heidelberg and after a one-week inpatient stay with total abstinence. Thereafter, most patients were released from the hospital. Alcohol intake and beverages consumed were assessed by the study nurses using a standardized questionnaire. All ALD patients enrolled in the present study were classified as heavy episodic drinkers (>60 g/alcohol per occasion per day [1]). Liver steatosis and signs of cirrhosis were determined by ultrasound (US) before alcohol withdrawal. In addition, liver stiffness before and after alcohol withdrawal was measured by transient elastography (Fibroscan; Echosens SA, Paris, France) [12]. A cut-off value of 8 kPa was set to separate low (F0-F2) and advanced (F3-F4) fibrosis in ALD patients as described by Mueller et al. [13]. Hepatic steatosis before and after alcohol withdrawal was measured using controlled attenuation parameter (CAP) on the FibroScan device [14]. Healthy normal-weight controls consumed <10 g (women) and <20 g (men) of ethanol per day, had no signs of liver disease as determined by blood parameters and ultrasound, and had no history of other chronic disease. Neither ALD patients nor controls were treated with antibiotics in the last 6 months or regularly consumed pre- or probiotics.

### 2.2. Clinical Measures

ALT (alaline aminotransferase), AST (aspartate aminotransferase) and γGT (γ glutamyltransferase) activities in fasting serum samples of ALD patients and controls were assessed by a routine laboratory analysis (Analysis Centre, University of Heidelberg and Ihr Labor 1220, Vienna, Austria).

### 2.3. Markers of Intestinal Permeability

Bacterial endotoxin levels in serum were determined using a limulus amebocyte lysate assay (Charles River, Ecully, France) as detailed previously [15]. Lipopolysaccharide binding protein (LBP) and zonulin levels in serum were assessed using a commercially available ELISA Kit (LBP: Szabo Scandic, Vienna, Austria; zonulin: Immundiagnostik AG, Bensheim, Germany) following the instructions of the manufacturer. Commercially available reporter gene assays with TLR2 and TLR4 transfected HEK293 cells were used to determine the levels of TLR2 and TLR4 ligands, respectively, e.g., lipopolysaccharides from gram-negative and lipoteichoic acid from gram-positive bacteria in serum following the instructions of the manufacturer (InvivoGen, Toulouse, France, Cat.Number: hTLR2= hkb-htlr2, hTLR4 = hkb-htlr4). In brief, cells were grown up to 80% confluence and challenged with 20 µl of serum for 12 h. Color changes of medium, being indicative of ligand concentration, were determined at 635 nm. Intestinal fatty acid binding protein (I-FABP) concentrations were detected in serum by western blot using a commercially available monoclonal anti-I-FABP antibody (Abcam, Cambridge, UK).

### 2.4. Statistical Analysis

Data are presented as means ± standard error of mean (SEM). Statistical analysis was performed using PRISM (version 7.03, GraphPad Software, Inc., San Diego, CA, USA). Grubb’s tests were used to determine outliers before statistical analysis. Shapiro-Wilk tests were applied to test for normality. Data were log-transformed if normality of data was not given. For comparison of data of ALD patients, a paired *t*-test was used. For comparison of data of healthy non-drinkers with ALD patients before and after alcohol withdrawal, a one-way ANOVA was performed and *p* ≤ 0.05 was selected as the level of significance.

## 3. Results

### 3.1. Characteristics of Controls and ALD Patients

Age and gender distribution were similar between ALD patients (*n* = 37) and controls (*n* = 17) whereas BMI of ALD patients was significantly higher compared to healthy controls (Table 1). As determined by ultrasound, 7 out of the 37 ALD patients showed signs of cirrhosis. In 34 out of the 37 ALD patients, steatosis ranged from 1 (*n* = 7) through 2 (*n* = 19) to 3 (*n* = 9). In 3 patients with ALD, hepatic steatosis was not assessed by ultrasound. Liver stiffness was elevated in ALD patients and was significantly lower after withdrawal while fibrosis stages were mostly unchanged. In line with the reduction of liver stiffness, hepatic fat was also significantly reduced during alcohol withdrawal (Table 1). ALD patients consumed on average 191 g/d ethanol (men: ~206 g/d; women: ~156 g/d) while controls consumed <10 g (women) and <20 g (men). Out of ALD patients stating to predominately consume beer (*n* = 17), five also reported to consume hard spirits, whereas of those reporting to drink wine (*n* = 17), four reported to consume hard spirits. Six of the ALD patients reported to only drink hard spirits. Average duration of alcohol consumption in ALD patients was 29.7 ± 1.9 years (Table 1). As expected, activities of ALT, AST, and γ-GT in serum were significantly higher in ALD patients than in controls before alcohol withdrawal (Table 1). After the alcohol withdrawal ALT, AST and γ-GT activities were significantly lower but were still significantly higher than in healthy controls (Table 1).

### 3.2. Markers of Intestinal Permeability in ALD Patients before and after Alcohol Withdrawal

**Figure 1:**
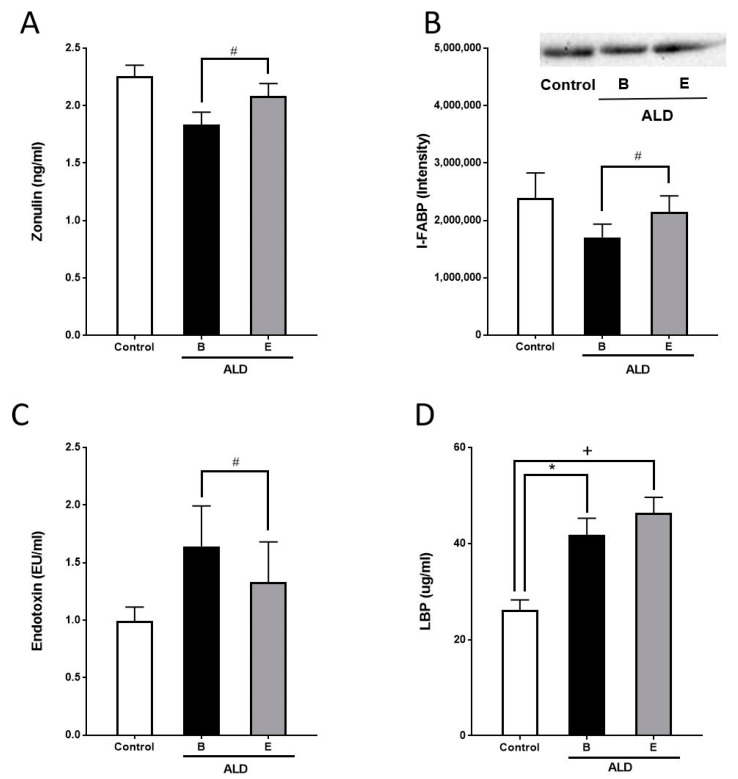
Serum markers of intestinal permeability in controls and patients with ALD before and after alcohol withdrawal. (**A**) Zonulin and (**B**) I-FABP protein concentrations, (**C**) endotoxin levels and (**D**) LBP protein concentration in serum of healthy controls and patients with ALD. ALD: alcohol-related liver disease, B: beginning of alcohol withdrawal, E: end of alcohol withdrawal, I-FABP: intestinal fatty acid binding protein, LBP: lipopolysaccharide binding protein. Data are shown as mean ± SEM. Levels of significance for: # *p* = 0.05, * *p* = 0.05, + *p* = 0.05.

Before alcohol withdrawal, I-FABP and zonulin protein concentrations in serum, having been suggested by others before to be indicative of intestinal barrier (dys-)function (for overview see [16]), were lower in ALD patients compared to healthy controls but differences did not reach the level of significance (zonulin: *p* = 0.06; I-FABP: *p* = 0.27, Figure 1). In contrast, bacterial endotoxin levels were higher in ALD patients than in controls, but as concentrations varied considerably among ALD patients, differences did not reach the level of significance (Figure 1). LBP protein concentrations in serum were significantly higher in ALD patients than in controls (Figure 1).

After the alcohol withdrawal, I-FABP and zonulin protein levels in serum of ALD patients were significantly increased almost to the level of controls (*p* < 0.05 for both parameters). Bacterial endotoxin serum levels were significantly lower after the alcohol withdrawal (*p* < 0.05) and almost reached the level of controls. LBP protein levels in serum of ALD patients were not altered by the alcohol withdrawal (Figure 1A–D).

**Figure 2:**
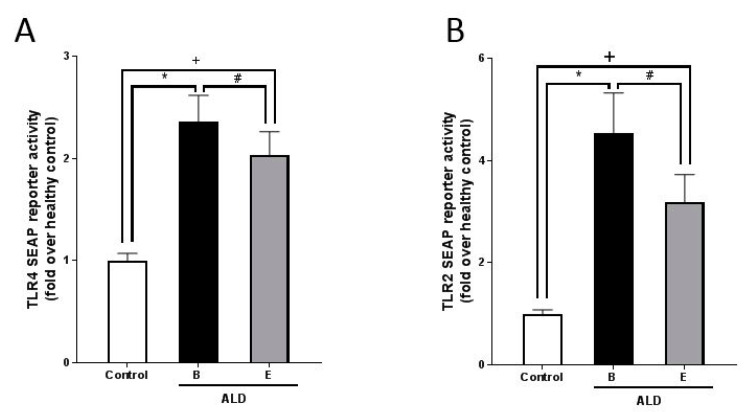
Concentration of TLR2 and TLR4 ligands in serum as determined by assessing activation of TLR4 and TLR2 transfected HEK293 cells, respectively, incubated with serum of controls and patients with ALD before and after alcohol withdrawal. SEAP reporter activity in HEK 293 cells transfected with (**A**) human TLR4 receptor and (**B**) human TLR2 receptor, respectively, challenged with serum of controls and patients with ALD. ALD: alcohol-related liver disease, B: beginning of alcohol withdrawal, E: end of alcohol withdrawal, SEAP: secreted embryonic alkaline phosphatase, TLR = toll-like receptor: Data are shown as mean ± SEM. Levels of significance for: # *p* = 0.05, * *p* = 0.05, + *p* = 0.05.

Activations of TLR2 and TLR4 transfected cells, respectively, being indicative of TLR2 and TLR4 ligand concentration in serum, were also significantly higher in ALD patients than in controls (Figure 2). After the alcohol withdrawal activation of both, TLR2 and TLR4 transfected HEK293 cells, were significantly lower (*p* < 0.05 for both); however, activation of both cell types was still higher than in controls (*p* < 0.05).

## 4. Discussion

Results of several human studies suggest that acute high and chronic intake of alcohol are associated with increased bacterial endotoxin levels and impairments of intestinal barrier function, and the development of ALD [4]. While results of some human studies suggest that total abstinence is associated with improvements of markers of intestinal barrier function [9], data assessing the effects of alcohol withdrawal in relation with the effects of abstinence on liver health are rather limited. In the present study, paralleling the results found regarding liver health, markers of intestinal barrier function, found to be markedly different from those of controls, gradually returned to the levels of controls. However, while transaminases were significantly higher before alcohol withdrawal, endotoxin levels in serum were only slightly elevated in ALD patients compared to controls. The lack of significance between controls and ALD patients might have resulted from the fact that endotoxin levels were not elevated in all ALD patients. This is in contrast with the findings of others reporting higher bacterial endotoxin levels in ALD patients [17,18]. In the present study, in 24 ALD patients, bacterial endotoxin levels were at the level of controls. While recovery rates in spiked serum samples of ALD patients were on average at 101.9 ± 6.4%, it could be that bacterial endotoxin was not detectable at least in part due to unspecific binding to serum proteins. Still, in line with the findings of others in patients with alcohol use disorder (AUD), endotoxin levels decrease during the week of alcohol withdrawal.

Furthermore, results of the reporter gene assays employing TLR4 and TLR2 transfected cells, respectively, suggest that ligands of TLR4 and TLR2 were present in serum samples in ALD patients in significantly higher amounts than in controls and also decreased during alcohol withdrawal. In line with these findings, LBP protein levels were also significantly higher in ALD patients than in controls; however, LBP protein levels were not affected by the alcohol withdrawal. LBP has suggested to be critical in mediating cellular effects of bacterial endotoxin levels through binding the toxin and transferring it to TLR4 [19]. It has been shown that the rise of LBP is relatively slow (peak concentrations 2–3 days after onset of acute phase (for overview see [20]). Therefore, it could be that one week was not sufficient to alter LBP serum concentration. Results of Turunen et al. also suggest that bacterial endotoxin levels are not always elevated in all heavy drinkers with ALD [21]. Furthermore, in the present study, protein levels of zonulin and I-FABP were both described as markers of intestinal barrier function [22,23] and were lower in serum of ALD patients before alcohol withdrawal than in controls and increased in the due course of alcohol withdrawal. This is in contrast to the findings of others in patients with alcohol used disorder (AUD) or healthy volunteers after acute alcohol ingestion showing increased levels of both zonulin and I-FABP, both having been suggested to be markers of intestinal permeability in comparison to healthy and sober controls [24,25]. Differences between our study and those of others might have resulted from the duration and severity of alcohol abuse as well as general health status, e.g., the presence of ALD in our study while other studied patients with AUD with unknown liver status. Both markers have also been suggested to not only reflect enterocyte damage but also mass and seem to be affected by cell turnover [26]. Furthermore, I-FABP has been suggested to be exclusively found in mature enterocytes in the upper half of the villous epithelium [26] and shown to be especially vulnerable to effects of ethanol [27]. However, further studies are needed to determine mechanisms involved in the decreased levels of I-FABP and zonulin found in the present study.

### Limitations

Our study is not without limitations. Indeed, a major limitation is the rather small samples size (37 ALD patients and 17 age-matched controls) of primarily Caucasian participants and the lack of longer follow-up periods (>1 week) and more frequent measurements. Therefore, it cannot be ruled out that results might differ in larger populations with mixed or other ethnic background. It cannot be ruled out that effects on markers of intestinal barrier might even further improve or not persist over time. However, despite the small sample size and the short follow-up, our findings are in line with others showing an association of the development of ALD and alterations of the intestinal barrier function, and an improvement of the latter parameters in the due course of the alcohol withdrawal of a longer period of time. Another major limitation is the lack of a negative control group composed of ALD patients not undergoing alcohol withdrawal. However, as it was not possible to enroll this group without accepting a major risk of bias (e.g., differences in environments including inpatient care vs. at home setting), we refrained from enrolling this patient group in the present study. All markers were also assessed at baseline so that each patient could serve as his/her own control. Another limitation is that ALD was not biopsy-proven but rather diagnosed by hepatic ultrasound, Fibroscan and the elevation of serum transaminase activity. However, for ethical reasons it was not possible to obtain liver biopsies from patients before and after alcohol withdrawal in the present study. Indeed, due to its non-invasiveness and safety as well as its accuracy and reliability to detect ALD, ultrasonography and Fibroscan were the techniques of choice [28]. Furthermore, for ethical and compliance reasons only, indirect markers of gut permeability, e.g., plasma endotoxin, I-FABP, zonulin and TLR2/4 reported gene assays, were used to determine intestinal barrier dysfunction. Still, findings are, in part, in line with those of others using PEG or lactulose/mannitol assays to determine intestinal permeability in ALD patients [29,30]. A further limitation is the fact that intestinal microbiota composition was not assessed.

## 5. Conclusions

Taken together, results of the present study further bolster the hypothesis that increased bacterial toxin levels are directly related to alcohol intake in ALD patients and that markers of intestinal barrier function are altered. Our results also suggest that even after only one week of abstinence these alterations are markedly improved, almost to the level of healthy controls and that this is associated with an improvement of liver healthiness. However, if these effects are primarily a result of a restored/healed intestinal mucosa or also related to an improved clearance of bacterial toxins and changes in intestinal microbiota, composition needs to be determined in future studies.

## Figures and Tables

**Table 1:** Characteristics and liver parameters of healthy non-drinkers (controls) and patients with alcohol-related liver disease (ALD) undergoing alcohol withdrawal.

Parameter	Controls	ALD Patients
Gender (m/f)	(10/7)	(26/11)
Age (years)	45.1 ± 2.3	49.0 ± 1.5
BMI (kg/m^2^)	22.7 ± 0.7	27.2 ± 1.09 ^a^
High alcohol consumption (years)	n.a.	29.7 ± 1.9
Alcohol (g/d) (m/f)	-	206.6 ± 25.0/156.0 ± 24.3
Steatosis	-	
Grade 1	0	7
Grade 2	0	19
Grade 3	0	8
unclassified	0	3
AST (U/L)		
before	21.1 ± 1.0	104.8 ± 14.7 ^a^
after	-	62.17 ± 4.8 ^a,b^
ALT (U/L)		
before	19.5 ± 0.8	78.0 ± 9.3 ^a^
after	-	66.47 ± 7.3 ^a,b^
gGT (U/L)		
before	17.5 ± 1.2	587.7 ± 132.7 ^a^
after	-	414.9 ± 94.6 ^a,b^
Liver stiffness		
Before (kPa)	-	17.42 ± 3.68
F0-F2 (*n*)	-	24
F3-F4 (*n*)	-	12
After (kPa)	-	13.86 ± 2.92 ^b^
F0-F2 (*n*)	-	22
F3-F4 (*n*)	-	14
CAP (dB/m)		
before	-	315.0 ± 7.7
after	-	274.9 ± 10.6 ^b^

BMI: body mass index, AST: aspartate aminotransferase, ALT: alanine aminotransferase, gGT: gamma glutamyltransferase, CAP: controlled attenuation parameter, n.a.: not applicable. Data are shown as mean ± SEM. ^a^ = *p* < 0.05 compared to healthy controls, ^b^ = *p* < 0.05 compared to ALD patients before alcohol withdrawal.

## References

[1] WHO (2018). Global Status Report on Alcohol and Health 2018.

[2] Seitz H.K., Bataller R., Cortez-Pinto H., Gao B., Gual A., Lackner C., Mathurin P., Mueller S., Szabo G., Tsukamoto H. (2018). Alcoholic liver disease. Nat. Rev. Dis. Primers.

[3] Mann R.E., Smart R.G., Govoni R. (2003). The epidemiology of alcoholic liver disease. Alcohol Res. Health.

[4] Hartmann P., Seebauer C.T., Schnabl B. (2015). Alcoholic liver disease: The gut microbiome and liver cross talk. Alcohol Clin. Exp. Res..

[5] Chen Y., Yang F., Lu H., Wang B., Chen Y., Lei D., Wang Y., Zhu B., Li L. (2011). Characterization of fecal microbial communities in patients with liver cirrhosis. Hepatology.

[6] Rivera C.A., Bradford B.U., Seabra V., Thurman R.G. (1998). Role of endotoxin in the hypermetabolic state after acute ethanol exposure. Am. J. Physiol..

[7] Yuki T., Thurman R.G. (1980). The swift increase in alcohol metabolism. Time course for the increase in hepatic oxygen uptake and the involvement of glycolysis. Biochem. J..

[8] Szabo G. (2015). Gut-Liver Axis in Alcoholic Liver Disease. Gastroenterology.

[9] Leclercq S., Cani P.D., Neyrinck A.M., Starkel P., Jamar F., Mikolajczak M., Delzenne N.M., de Timary P. (2012). Role of intestinal permeability and inflammation in the biological and behavioral control of alcohol-dependent subjects. Brain Behav. Immun..

[10] De Ledinghen V., Vergniol J., Capdepont M., Chermak F., Hiriart J.B., Cassinotto C., Merrouche W., Foucher J., le Brigitte B. (2014). Controlled attenuation parameter (CAP) for the diagnosis of steatosis: A prospective study of 5323 examinations. J. Hepatol..

[11] Mueller S., Nahon P., Rausch V., Peccerella T., Silva I., Yagmur E., Straub B.K., Lackner C., Seitz H.K., Rufat P.A. (2017). Caspase-cleaved keratin-18 fragments increase during alcohol withdrawal and predict liver-related death in patients with alcoholic liver disease. Hepatology.

[12] Mueller S. (2020). Liver Elastography: Clinical Use and Interpretation.

[13] Mueller S., Seitz H.K., Rausch V. (2014). Non-invasive diagnosis of alcoholic liver disease. World J. Gastroenterol..

[14] Thiele M., Rausch V., Fluhr G., Kjaergaard M., Piecha F., Mueller J., Straub B.K., Lupsor-Platon M., De-Ledinghen V., Seitz H.K. (2018). Controlled attenuation parameter and alcoholic hepatic steatosis: Diagnostic accuracy and role of alcohol detoxification. J. Hepatol..

[15] Sellmann C., Baumann A., Brandt A., Jin C.J., Nier A., Bergheim I. (2017). Oral Supplementation of Glutamine Attenuates the Progression of Nonalcoholic Steatohepatitis in C57BL/6J Mice. J. Nutr..

[16] Tajik N., Frech M., Schulz O., Schalter F., Lucas S., Azizov V., Durholz K., Steffen F., Omata Y., Rings A. (2020). Targeting zonulin and intestinal epithelial barrier function to prevent onset of arthritis. Nat. Commun..

[17] Bode C., Kugler V., Bode J.C. (1987). Endotoxemia in patients with alcoholic and non-alcoholic cirrhosis and in subjects with no evidence of chronic liver disease following acute alcohol excess. J. Hepatol..

[18] Fukui H., Brauner B., Bode J.C., Bode C. (1991). Plasma endotoxin concentrations in patients with alcoholic and non-alcoholic liver disease: Reevaluation with an improved chromogenic assay. J. Hepatol..

[19] Dentener M.A., von Asmuth E.J., Francot G.J., Marra M.N., Buurman W.A. (1993). Antagonistic effects of lipopolysaccharide binding protein and bactericidal/permeability-increasing protein on lipopolysaccharide-induced cytokine release by mononuclear phagocytes. Competition for binding to lipopolysaccharide. J. Immunol..

[20] Schumann R.R. (2011). Old and new findings on lipopolysaccharide-binding protein: A soluble pattern-recognition molecule. Biochem. Soc. Trans..

[21] Turunen U., Malkamaki M., Valtonen V.V., Larinkari U., Pikkarainen P., Salaspuro M.P., Makela P.H. (1981). Endotoxin and liver diseases. High titres of enterobacterial common antigen antibodies in patients with alcoholic cirrhosis. Gut.

[22] Lau E., Marques C., Pestana D., Santoalha M., Carvalho D., Freitas P., Calhau C. (2016). The role of I-FABP as a biomarker of intestinal barrier dysfunction driven by gut microbiota changes in obesity. Nutr. Metab. Lond..

[23] Kim J.H., Heo J.S., Baek K.S., Kim S.Y., Kim J.H., Baek K.H., Kim K.E., Sheen Y.H. (2018). Zonulin level, a marker of intestinal permeability, is increased in association with liver enzymes in young adolescents. Clin. Chim. Acta.

[24] De Jong W.J., Cleveringa A.M., Greijdanus B., Meyer P., Heineman E., Hulscher J.B. (2015). The effect of acute alcohol intoxication on gut wall integrity in healthy male volunteers; a randomized controlled trial. Alcohol.

[25] Donnadieu-Rigole H., Pansu N., Mura T., Pelletier S., Alarcon R., Gamon L., Perney P., Apparailly F., Lavigne J.P., Dunyach-Remy C. (2018). Beneficial Effect of Alcohol Withdrawal on Gut Permeability and Microbial Translocation in Patients with Alcohol Use Disorder. Alcohol Clin. Exp. Res..

[26] Derikx J.P., Blijlevens N.M., Donnelly J.P., Fujii H., Kanda T., van Bijnen A.A., Heineman E., Buurman W.A. (2009). Loss of enterocyte mass is accompanied by diminished turnover of enterocytes after myeloablative therapy in haematopoietic stem-cell transplant recipients. Ann. Oncol..

[27] Cho Y.E., Yu L.R., Abdelmegeed M.A., Yoo S.H., Song B.J. (2018). Apoptosis of enterocytes and nitration of junctional complex proteins promote alcohol-induced gut leakiness and liver injury. J. Hepatol..

[28] Moreno C., Mueller S., Szabo G. (2019). Non-invasive diagnosis and biomarkers in alcohol-related liver disease. J. Hepatol..

[29] Keshavarzian A., Holmes E.W., Patel M., Iber F., Fields J.Z., Pethkar S. (1999). Leaky gut in alcoholic cirrhosis: A possible mechanism for alcohol-induced liver damage. Am. J. Gastroenterol..

[30] Parlesak A., Schafer C., Schutz T., Bode J.C., Bode C. (2000). Increased intestinal permeability to macromolecules and endotoxemia in patients with chronic alcohol abuse in different stages of alcohol-induced liver disease. J. Hepatol..

